# Thriving-in-Place: Examining the impact of intergenerational living in the Toronto HomeShare Program

**DOI:** 10.1093/geroni/igab046.3748

**Published:** 2021-12-17

**Authors:** Raza Mirza, Melissa Macri, Deirdre Kelly-Adams, Carley Moore, Andrea Austen, Jacalyn Tanner, Jessica Hsieh, Christopher Klinger

**Affiliations:** 1 University of Toronto, Toronto, Ontario, Canada; 2 City of Toronto, City of Toronto, Ontario, Canada; 3 National Initiative for the Care of the Elderly (NICE), Toronto, Ontario, Canada

## Abstract

Most global cities, like Toronto, have rapidly aging populations who want to remain in homes and communities of their choice. Concurrently, seniors face vulnerabilities associated with low income, ageism, social isolation and loneliness. These vulnerabilities inhibit many seniors’ desires to age-in-place. The Toronto HomeShare Program, an intergenerational homesharing program facilitates aging-in-place by matching seniors with post-secondary students. The program, with an implementation focus and a research study, was developed to address and understand the needs of seniors seeking assistance, light supports and companionship at home, in exchange for reduced-rent housing for students. A mixed methods research design was employed. Seniors and students (n=22) completed a 167 question survey (n=22) and in-depth interviews (n=18). Quantitative data yielded descriptive statistics and qualitative data was subject to thematic content analysis. Participants agreed that homesharing programs could address risk for social isolation (95%), the need to move from their community (96%), and reduce risks of economic and social exclusion for young and old (97%). From the qualitative data, six benefits were apparent for all participants: (1) reduced social isolation and loneliness, (2) increased intergenerational exchange, (3) increase financial security, (4) household assistance, (5) increased general wellbeing; (6) enhanced companionship/safety. In 2020, Toronto HomeShare (now Canada HomeShare) was recognized by the World Health Organization as an age-friendly best practice, and has been scaled nationally in 16 cities. Intergenerational homesharing programs could be a catalyst for policy and cultural reform and to support older adults to not only remain in their communities, but to thrive-in-place.

